# Factors Associated With the Use of Digital Technology Among Youth in Zimbabwe: Findings From a Cross-Sectional Population-Based Survey

**DOI:** 10.2196/52670

**Published:** 2024-09-23

**Authors:** Kevin Martin, Rachel Wei Chun Peh, Mandikudza Tembo, Constancia Vimbayi Mavodza, Aoife M Doyle, Chido Dziva Chikwari, Ethel Dauya, Tsitsi Bandason, Steven Azizi, Victoria Simms, Rashida A Ferrand

**Affiliations:** 1 Department of Clinical Research London School of Hygiene & Tropical Medicine London United Kingdom; 2 The Health Research Unit Zimbabwe Biomedical Research and Training Institute Harare Zimbabwe; 3 MRC International Statistics and Epidemiology Group London School of Hygiene & Tropical Medicine London United Kingdom; 4 Department of Global Health and Development Faculty of Public Health and Policy London School of Hygiene & Tropical Medicine London United Kingdom; 5 Department of Public Health, Environments and Society London School of Hygiene & Tropical Medicine London United Kingdom

**Keywords:** Zimbabwe, youth, digital technology, household wealth

## Abstract

**Background:**

Globally, the increasing use of digital technologies such as mobile phones and the internet has allowed for the development of innovative mobile health interventions, particularly for reaching and engaging with youth. However, there is a risk that using such technologies may exclude those who lack access to them.

**Objective:**

In this study, we investigated the sociodemographic factors associated with mobile phone ownership, internet use, and social media use among youth in Zimbabwe.

**Methods:**

A population-based prevalence survey was conducted in 24 urban and periurban communities across 3 provinces of Zimbabwe (Harare, Mashonaland East, and Bulawayo). Youths aged 18 to 24 years resident in randomly selected households in the study communities completed an interviewer-administered questionnaire. The primary outcomes were mobile phone ownership and current internet and social media use. A household wealth indicator was developed using principal components analysis, based on household asset ownership. Multivariable logistic regression was used to investigate the factors associated with each primary outcome. Age, sex, and province were considered a priori confounders. Household wealth, marital status, education level, employment status, time lived at current address, and HIV status were included in the final multivariable model if there was an age-, sex-, and province-adjusted association with a primary outcome on univariable analysis at a significance level of *P*<.10.

**Results:**

Of the 17,636 participants assessed for the primary outcome, 16,370 (92.82%) had access to a mobile phone, and 15,454 (87.63%) owned a mobile phone. Among participants with access to a mobile phone, 58.61% (9594/16,370) and 57.79% (9460/16,370), respectively, used internet and social media at least weekly. Older age (adjusted odds ratio [aOR] 1.76, 95% CI 1.55-2.00), increasing wealth (ranging from aOR 1.85, 95% CI 1.58-2.16, for wealth quintile 2 to aOR 3.80, 95% CI 3.00-4.80, for wealth quintile 5, with quintile 1 as reference), and higher education level (secondary: aOR 1.96, 95% CI 1.60-2.39; tertiary: aOR 8.36, 95% CI 5.29-13.20) were associated with mobile phone ownership. Older age, male sex, increasing wealth, having never been married, higher education level, being in education or formal employment, and having lived at the same address for ≥2 years were associated with higher levels of internet and social media use.

**Conclusions:**

While mobile phone ownership was near-universal, over one-third of youths in urban and periurban settings did not have access to the internet and social media. Access to the internet and social media use were strongly associated with household wealth and education level. Mobile health interventions must ensure that they do not amplify existing inequalities in access to health care. Such interventions must be accompanied by alternative strategies to engage and enroll individuals without internet or social media access to prevent the exclusion of young people by sex and socioeconomic status.

## Introduction

### Background

Mobile phone use is rapidly increasing in many countries in the Global South, where this is expanding faster than other infrastructures [1]. Mobile technology is increasingly being deployed to support health care and improve access to public health interventions, including health promotion and facilitating engagement with and access to particular health services [2]. Mobile health (mHealth) may be implemented through mobile phones, internet access, and social media. mHealth approaches range from simple reminders via text message to improve medication compliance [3], locating study participants or delivery of health messaging through social media or other applications [4,5], to more complex methods such as wearable technologies [6]. This transition to increasing use of digital health technologies has been accelerated by the COVID-19 pandemic, when use of such technologies was essential to deliver public health interventions and maintain routine services that could not be delivered face to face [7].

Digital technologies may be particularly suitable for young people, who have grown up in an age where mobile phone use is largely normalized and where access and availability to the internet is higher than before [1]. However, we must recognize that the distribution of mobile phone ownership, including smart phone ownership, and internet connectivity is not equal, neither across nor within countries [1,8]. Data from the International Telecommunication Union show that in 2022, although almost all young people (aged 15-24 years) in high-income countries had access to the internet, only 67% and 39% in lower–middle-income and low-income countries, respectively, used the internet [9]. The *Lancet* and Financial Times Commission on governing health futures 2030 has also reported that although internet uptake is growing globally, the digital divide between sexes is also widening, which may exacerbate the existing inequalities [1]. Although there has been significant growth in mobile technologies globally, a 2018 survey across 18 “advanced” economies and 9 “emerging” economies found that a median of 76% and 45% of respondents within each category owned a smartphone, respectively [8].

Disparities in mobile phone use are also mirrored in the mHealth literature. Although the number of studies assessing the effectiveness of mHealth interventions in promoting health outcomes or modifying behavior is growing globally, with an overall positive albeit weak effect demonstrated, data are still very sparse for the Global South [10,11]. A 2021 systematic review of studies assessing mHealth apps for health promotion and disease management found 172 studies meeting their inclusion criteria. However, only 8 studies were performed outside of high-income settings [10]. One particular example of an mHealth intervention in Zimbabwe is a digital mental health intervention called “Inuka,” delivered via a chat-based application, which demonstrated high levels of acceptability, feasibility, and appropriateness [12]. However, the barriers to the use of the app and to potential integration into routine care, did include challenges with connectivity, which negatively affected some of the clients’ experiences. Given the poorer connectivity in high-density suburbs and rural areas than in low-density suburbs and urban areas, the authors noted that this would affect the feasibility of delivering an equitable rollout of such an intervention in this setting [13].

Due to the heterogeneity in the distribution of mobile technology and of ownership and use across settings, it is critical to understand the context and digital landscape before digital technologies are deployed, as there is potential for these to amplify inequalities [12]. Exploring access to mobile phone ownership, internet access, and social media use is necessary to understand who would and would not potentially benefit from particular mHealth interventions. Furthermore, it would allow for the barriers to be preempted and for alternate strategies to be developed. This is particularly pertinent for Zimbabwe, where data on technology use are limited. The 2015 Zimbabwe Demographic and Health Survey reported that 87% of households owned a mobile phone [14]. However, this does not necessarily mean that young people within these households have access. This is demonstrated by Doyle et al [15] who conducted a relatively small survey of young people aged 13 to 24 years in urban communities in Zimbabwe and found that 63% of the young people surveyed owned a mobile phone and 65% had access to the internet [15]. More data are needed to confirm these findings and to further explore factors associated with technology use.

### Objective

The objectives of this study were to investigate the levels of mobile phone ownership, internet use, and social media use and to understand the factors associated with mobile phone ownership, internet use, and social media use among youth in Zimbabwe.

## Methods

### Study Design and Setting

The study used data from a cross-sectional population-based survey conducted to ascertain the outcome of a cluster randomized trial investigating the impact of providing a community-based package of integrated HIV and sexual and reproductive health services on population-level HIV outcomes. The study protocol with details of the intervention and the survey has been previously published [16].

Briefly, the 2-arm CHIEDZA trial (*Community-based interventions to improve HIV outcomes in youth: a cluster randomised trial in Zimbabwe*; trial registration NCT03719521) was conducted in 24 communities in 3 provinces (Harare, Mashonaland East and Bulawayo), with each province containing 8 clusters randomized 1:1 to 4 intervention and 4 standard of care (routine, existing services) clusters. A *cluster* was defined as a geographically demarcated area in a community containing a primary health care clinic and a community center from which services are delivered. The 3 provinces were chosen to include areas representing both main ethnic groups (Bulawayo is predominantly Ndebele, and Harare and Mashonaland East are predominantly Shona) and to include both urban and periurban settings. Rural settings were excluded, as population density was too low to make the delivery of the CHIEDZA intervention feasible [16]. Individuals aged 16 to 24 years living within an intervention cluster were eligible to receive a package of integrated HIV and sexual and reproductive health services delivered from the intervention cluster community center. This package included HIV testing, HIV treatment and adherence support, contraception, pregnancy testing, syndromic management of sexually transmitted infections, menstrual health information and products, condoms, and general health counseling in addition to existing health services (standard of care). Service delivery was accompanied by peer outreach to promote CHIEDZA and engage youth to achieve high coverage. Outreach teams consisted of 16-to 24-year-old cluster residents who had previously engaged with CHIEDZA. Outreach activities included flyer distribution, information dissemination, and in-field live demonstrations of CHIEDZA products (such as reusable pads, menstrual cups, and condoms), alongside door-to-door sensitization within the cluster [16].

### Study Procedures

The postintervention prevalence survey was conducted after a 30-month intervention period, between October 2021 and June 2022, in both intervention and control clusters. Mapping of the streets within clusters was conducted by checking the OpenStreetMap road network against satellite imagery to ensure all streets were mapped. The streets were manually split into segments with ArcGIS software (Esri) using either junctions or features such as school grounds. Each segment was assigned a number, and segments were randomly selected. Following community sensitization, all *households* (defined as a person or group of related or unrelated persons who live together in the same dwelling or units of a dwelling, who acknowledge 1 adult male or female as the head of the household, who share the same housekeeping arrangements, and who are considered a single unit) in each dwelling in the randomly selected street segments were enumerated. All individuals aged 18 to 24 years residing in the enumerated households were eligible to participate. If a potentially eligible individual was not available at the time of enumeration, up to 3 repeat visits were made to enroll the individual. Following consent, data were collected using an interviewer-administered questionnaire, using SurveyCTO (Dobility Inc) on tablets. The survey was piloted in youth before being used in the prevalence survey. The relevant survey questions and response categories for this study are shown in Multimedia Appendix 1.

### Outcomes and Statistical Analysis

Sample size calculations for the survey were based on having sufficient power to ascertain the primary outcome of the CHIEDZA trial and are described in the trial protocol [16]. STATA (version 17.0; StataCorp) was used for data analysis. The primary outcomes in this study were mobile phone ownership, internet use, and social media use. *Mobile phone ownership* was defined as participants who have their own mobile phone, therefore excluding those who share a phone. *Internet use* was defined as participants who use the internet at least once per week on any device, for any reason (including social media). *Social media use* was defined as use of at least one of Twitter, Facebook, Instagram, TikTok, LinkedIn, YouTube, or Snapchat. WhatsApp was excluded from the internet use and social media definitions, as its function is largely similar to text messaging. In Zimbabwe, stand-alone WhatsApp data bundles that exclude internet use are much more affordable than either an internet connection or other data packages. Access to both the internet and social media will be largely influenced by access to a mobile phone [15]. As a result, internet use and social media use were only examined in participants who either owned or had access to a mobile phone. Secondary outcome variables included mobile phone functions (call, text, WhatsApp, internet browsing); frequency of internet used; internet access point; and types of social media platform used.

Principal component analysis was used to develop a household wealth indicator, based on the presence of 6 functioning household assets (fridge, bicycle, car or truck, television, radio, microwave; Multimedia Appendix 2). Univariable logistic regression was conducted to examine associations between variables and each primary outcome. For multivariable logistic regression, age, sex, and province were considered as a priori confounders. Other variables with an age-, sex-, and province-adjusted association with a primary outcome with a *P* value of <.10 were included in the final multivariable model. Clustering was adjusted for at the street segment level.

### Ethical Considerations

Ethical approval was obtained from the Medical Research Council of Zimbabwe (MRCZ/A/2387), the Biomedical Research and Training Institute Institutional Review Board (AP149/2018), and the London School of Hygiene and Tropical Medicine Ethics Committee (16,948). Consent was documented electronically on a tablet, with participants retaining a signed paper copy for their records.

## Results

### Participant Characteristics

**Figure 1:**
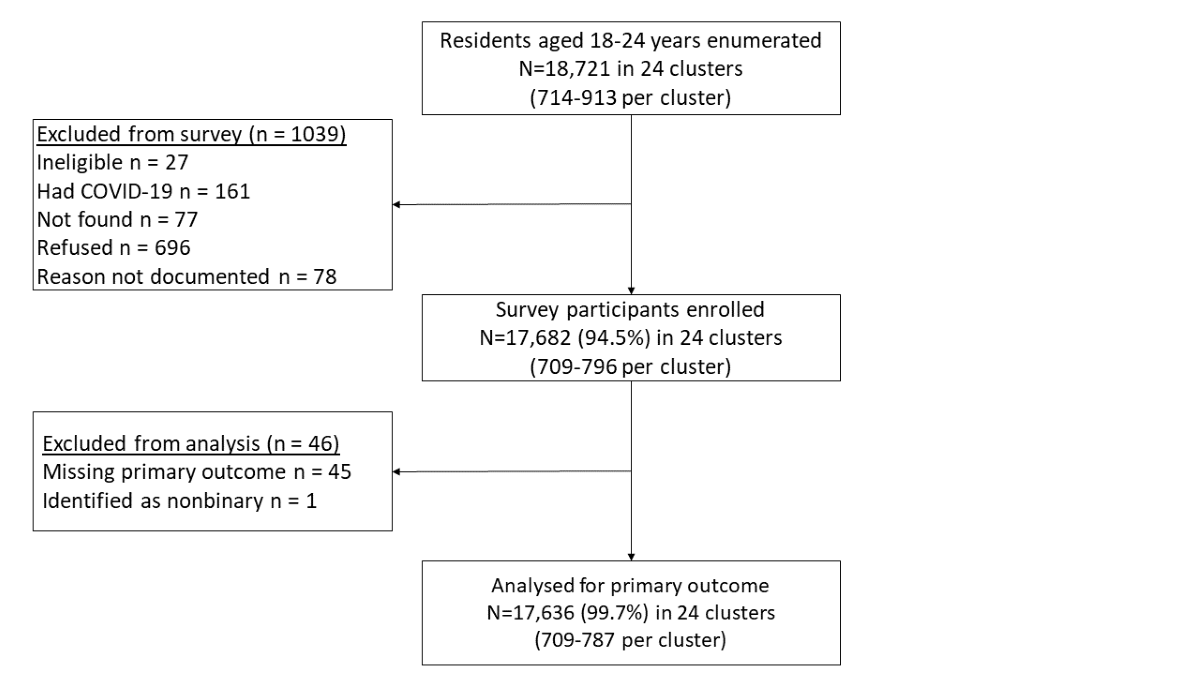
CONSORT (Consolidated Standards for Reporting Trials) diagram showing study recruitment of youth aged 18 to 24 years in a population-based survey in Zimbabwe to ascertain the outcome of a cluster randomized trial (community-based interventions to improve HIV outcomes in youth: a cluster randomised trial in Zimbabwe [CHIEDZA]).

The CONSORT (Consolidated Standards for Reporting Trials) diagram is shown in Figure 1. Of the 18,721 enumerated individuals, 17,682 (94.45%) were enrolled, of whom 17,636 (99.71%) were assessed for the primary outcome, with approximately one-third recruited from each province. Overall, participants had a mean age of 20.6 (SD 2.1) years, and 60.82% (n=10,726) were female. Three-quarters of the participants (n=13,279, 75.29%) had never been married, almost half (8711/17,636, 49.39%) were neither in employment nor education, and 87.11% (15,363/17,636) of the participants’ highest education level was secondary (Table 1).

**Table 1:** Characteristics of youth in Zimbabwe who were recruited into the population-based survey (n=17,636 unless otherwise stated).

Variable		Value, n (%)
**Sex**		
	Female	10,726 (60.82)
	Male	6910 (39.18)
**Age (y)**		
	18-20	9239 (52.39)
	21-24	8397 (47.61)
**Province**		
	Harare	5845 (33.14)
	Bulawayo	5929 (33.62)
	Mashonaland East	5862 (33.24)
**Household wealth index (n=17** **,** **628)**		
	1 (poorest)	4161 (23.6)
	2	3430 (19.46)
	3	3658 (20.75)
	4	3664 (20.79)
	5 (richest)	2715 (15.4)
**Marital status**		
	Never married	13,279 (75.29)
	Married or living together as if married	3559 (20.18)
	Divorced, widowed, or separated (and currently unmarried)	798 (4.52)
**Highest completed education level**		
	None or primary level	867 (4.92)
	Form 1-4	12,638 (71.66)
	Form 5-6	2725 (15.45)
	Tertiary level	1406 (7.97)
**Employment status**		
	In education	4962 (28.14)
	Formal employment	832 (4.72)
	Informal employment	3151 (17.87)
	Not in education or employment	8711 (49.39)
**Time lived at current address (years)**		
	<1	4241 (24.05)
	1-2	1701 (9.65)
	2-3	1685 (9.55)
	>3	10,009 (56.75)
**Previous residence**		
	Always lived at this address	6866 (38.93)
	Same suburb but different address	3747 (21.25)
	Same town or city but different suburb	3748 (21.25)
	Lived outside this town or city	3275 (18.57)
**Average monthly household income (US $)**		
	<50	2616 (14.83)
	50-100	4457 (25.27)
	101-200	4740 (26.88)
	201-500	2697 (15.29)
	501-900	436 (2.47)
	>900	157 (0.89)
	Don’t know or don’t want to say	2533 (14.36)
**HIV status (n=17** **,** **503)**		
	Positive	1190 (6.8)
	Negative	16,313 (93.2)

### Use of Digital Technologies

Of the 17,636 participants, 16,370 (92.82%) had access to a mobile phone, with 15,454 (87.63%) owning a mobile phone, 842 (4.77%) sharing a phone with a family member, and 74 (0.42%) sharing a phone with someone other than a family member. Among mobile phone owners, 98.89% (15,282/15,454), 97.75% (15,106/15,454), 86.51% (13,369/15,454), and 63.55% (9821/15,454) of mobile phones were equipped to call, text, use WhatsApp, and browse the internet, respectively.

Among participants (n=16,370) with access to a mobile phone, 58.61% (n=9594) reported internet use. Frequency of internet use ranged from never (n=6776, 41.39%), once or twice a week (n=4004, 24.46%), most days of the week (n=1969, 12.03%), every day (n=3086, 18.85%), to several times per day (n=535, 3.27%). Among internet users, this was most often on a personal phone (9039/9594, 94.22%), as opposed to on someone else’s phone or using a different type of device.

Among participants with mobile phone access (n=16,370), social media use was 57.79% (n=9460). The most accessed types of social media, in descending order, were Facebook (n=8785, 53.67%), Instagram (n=4396, 26.85%), YouTube (n=4315, 26.36%), TikTok (n=2857, 17.45%), Twitter (n=2257, 13.79%), and LinkedIn (n=583, 3.56%). WhatsApp was used regularly by 85.85% (n=14,054) of participants with mobile access.

Among participants who owned a WhatsApp-enabled phone, 98.62% (13,185/13,369) accessed WhatsApp regularly (Table S1 in Multimedia Appendix 3). For participants who owned an internet-enabled phone, 85.17% (8365/9821) reported internet use (Table S2 in Multimedia Appendix 3), in comparison to 17.34% (977/5633) of those who owned a mobile phone not equipped to access the internet.

### Factors Associated With Mobile Phone Ownership, Internet Use, and Social Media Use

Univariable associations with mobile phone ownership, internet use, and social media use are shown in Tables 2, 3, and 4, respectively. Multimedia Appendix 4 also shows each primary outcome by age, education level, and duration of residence, disaggregated to a higher degree than shown in Tables 2-4.

Higher wealth index, being unmarried, higher education level, being in education or formal employment, having lived at their current address for at least 2 years, having always lived at their address, not living with HIV, and being in Harare (compared to Mashonaland East) were all associated with higher levels of mobile phone ownership, internet use, and social media use. Being aged 21 to 24 years (compared to 18-20 years) was associated with mobile phone ownership and internet use, but not with social media use, whereas male sex was associated with internet and social media use, but not with mobile phone ownership.

In multivariable analysis, mobile phone ownership was associated with being aged 21 to 24 (adjusted odds ratio [aOR] 1.76, 95% CI 1.55-2.00), being in Harare (capital city) compared to both Bulawayo (aOR 0.88, 95% CI 0.76-1.01) and Mashonaland East (aOR 0.81, 95% CI 0.71-0.96), increasing household wealth, and increased education level (Table 2).

Internet use was associated with being aged 21 to 24 years (aOR 1.21, 95% CI 1.11-1.33), male sex (aOR 1.43, 95% CI 1.30-1.57), increasing household wealth, having never been married, increasing education level, being in education (aOR 2.19, 95% CI 1.97-2.44) or formal employment (aOR 1.55, 95% CI 1.26-1.89), having lived at their current address for >2 years (aOR 1.35, 95% CI 1.21-1.51), and having always lived at their current address (Table 3).

Social media use was associated with being aged 21 to 24 years (aOR 1.39, 95% CI 1.27-1.52), male sex (aOR 1.41, 95% CI 1.29-1.55), being in Harare compared to both Bulawayo (aOR 0.76, 95% CI 0.67-0.85) and Mashonaland East (aOR 0.82, 95% CI 0.72-0.92), increasing household wealth, having never been married, increasing education level, being in education (aOR 1.97, 95% CI 1.77-2.20) or formal employment (aOR 1.62, 95% CI 1.35-1.95), having lived at their current address for >2 years (aOR 1.21, 95% CI 1.09-1.35), and having always lived at their current address (Table 4).

**Table 2:** Univariable and multivariable associations between exposure variables and mobile phone ownership among youth recruited into a population-based survey in Zimbabwe (n=17,636).

Variable			Mobile phone ownership, n (%)		Unadjusted OR^a^ (95% CI)		Age-, sex-, and province-adjusted OR (95% CI)		Final adjusted OR (95% CI)^b^
**Age (y)**					*P*<.001		*P*<.001		*P*<.001
	18-20	7841/9239 (84.87)		1.00		1.00		1.00	
	21-24	7613/8397 (90.66)		1.77 (1.59-1.97)		1.78 (1.60-1.99)		1.76 (1.55-2.00)	
**Sex**					*P*=.24		*P*=.09		*P*=.10
	Female	9364/10,726 (87.3)		1.00		1.00		1.00	
	Male	6090/6910 (88.13)		1.08 (0.95-1.22)		1.11 (0.98-1.26)		0.90 (0.79-1.02)	
**Province**					*P*<.001		*P*<.001		*P*=.03
	Harare	5227/5845 (89.42)		1.00		1.00		1.00	
	Bulawayo	5231/5929 (88.23)		0.92 (0.79-1.06)		0.95 (0.82-1.09)		0.88 (0.76-1.01)	
	Mashonaland East	4996/5862 (85.23)		0.66 (0.56-0.77)		0.67 (0.57-0.79)		0.83 (0.71-0.96)	
**Household wealth index**					*P*<.001		*P*<.001		*P*<.001
	1 (poorest)	3274/4161 (78.68)		1.00		1.00		1.00	
	2	2994/3430 (87.29)		1.97 (1.69-2.30)		2.00 (1.71-2.33)		1.85 (1.58-2.16)	
	3	3267/3658 (89.31)		2.51 (2.11-2.98)		2.57 (2.16-3.05)		2.30 (1.92-2.75)	
	4	3371/3664 (92)		3.36 (2.82-4.00)		3.49 (2.93-4.16)		3.04 (2.53-3.66)	
	5 (richest)	2541/2715 (93.59)		4.46 (3.57-5.56)		4.64 (3.71-5.80)		3.80 (3.00-4.80)	
**Marital status**					*P*<.001		*P*<.001		*P*=.47
	Never married	11,737/13,279 (88.39)		1.00		1.00		1.00	
	Married or living together as if married	3011/3559 (84.6)		0.77 (0.68-0.87)		0.61 (0.52-0.71)		0.93 (0.78-1.11)	
	Divorced, widowed, or separated (and currently unmarried)	706/798 (88.47)		1.01 (0.78-1.32)		0.80 (0.61-1.06)		1.10 (0.82-1.47)	
**Highest completed education level**					*P*<.001		*P*<.001		*P*<.001
	None or primary level	647/867 (74.63)		1.00		1.00		1.00	
	Secondary level	13,432/15,363 (87.43)		2.26 (1.87-2.72)		2.36 (1.94-2.86)		1.96 (1.60-2.39)	
	Tertiary level	1375/1406 (97.8)		17.3 (11.00-27.06)		14.98 (9.52-23.58)		8.36 (5.29-13.20)	
**Employment status**					*P*<.001		*P*<.001		*P*<.001
	Not in education or employment	7372/8711 (84.63)		1.00		1.00		1.00	
	Informal employment	2831/3151 (89.84)		1.71 (1.48-1.99)		1.58 (1.34-1.85)		1.54 (1.31-1.81)	
	In education	4462/4942 (90.29)		1.63 (1.43-1.87)		1.81 (1.57-2.08)		1.20 (1.03-1.39)	
	Formal employment	789/832 (94.83)		3.64 (2.62-5.05)		3.13 (2.24-4.37)		2.30 (1.65-3.21)	
**Time lived at current address (y)**					*P*<.001		*P*=.001		*P*=.58
	<2	5099/5942 (85.81)		1.00		1.00		1.00	
	>2	10,355/11,694 (88.55)		1.28 (1.15-1.42)		1.24 (1.11-1.38)		1.04 (0.91-1.17)	
**Previous residence**					*P*<.001		*P*<.001		*P*=.01
	Always lived at this address	6119/6866 (89.12)		1.00		1.00		1.00	
	Same suburb but different address	3260/3747 (87)		0.81 (0.70-0.94)		0.81 (0.70-0.94)		1.01 (0.86-1.19)	
	Same town or city but different suburb	3295/3748 (87.91)		0.92 (0.78-1.08)		0.93 (0.79-1.09)		1.11 (0.93-1.32)	
	Lived outside this town or city	2780/3275 (84.89)		0.67 (0.58-0.77)		0.69 (0.60-0.81)		0.83 (0.70-0.98)	
**HIV status**					*P*=.02		*P*=.004		*P*=.17
	Negative	14,327/16,313 (87.83)		1.00		1.00		1.00	
	Positive	1007/1190 (84.62)		0.79 (0.64-0.96)		0.73 (0.59-0.90)		0.85 (0.68-1.07)	

^a^OR: odds ratio.

^b^Adjusted for age, sex, province, household wealth, marital status, highest completed education level, employment status, and time lived at current address and previous residence.

**Table 3:** Univariable and multivariable associations between exposure variables and internet use among youth recruited into a population-based survey in Zimbabwe (n=16,370).

Variable		Internet use, n (%)	Unadjusted OR^a^ (95% CI)	Age-, sex-, and province-adjusted OR (95% CI)	Final adjusted OR (95% CI)^b^
**Age (y)**			*P*=.002	*P*=.07	*P*<.001
	18-20	5031/8423 (59.73)	1.00	1.00	1.00
	21-24	4563/7947 (57.42)	0.89 (0.83-0.96)	0.93 (0.87-1.01)	1.21 (1.11-1.33)
**Sex**			*P*<.001	*P*<.001	*P*<.001
	Female	5138/9978 (51.49)	1.00	1.00	1.00
	Male	4456/6392 (69.71)	2.05 (1.89-2.24)	2.05 (1.89-2.23)	1.43 (1.30-1.57)
**Province**			*P*<.001	*P*<.001	*P*=.06
	Harare	3189/5557 (57.39)	1.00	1.00	1.00
	Bulawayo	3548/5554 (63.88)	1.26 (1.11-1.43)	1.18 (1.04-1.34)	0.95 (0.83-1.09)
	Mashonaland East	2857/5259 (54.33)	0.77 (0.66-0.89)	0.73 (0.62-0.85)	0.84 (0.72-0.97)
**Household wealth index**			*P*<.001	*P*<.001	*P*<.001
	1 (poorest)	1367/3622 (37.74)	1.00	1.00	1.00
	2	1677/3196 (52.47)	1.92 (1.68-2.18)	1.81 (1.58-2.06)	1.53 (1.33-1.75)
	3	2152/3438 (62.59)	2.88 (2.52-3.28)	2.63 (2.31-3.00)	2.02 (1.76-2.32)
	4	2452/3497 (70.12)	4.19 (3.65-4.82)	3.84 (3.35-4.40)	2.64 (2.30-3.04)
	5 (richest)	1943/2610 (74.44)	5.23 (4.44-6.16)	4.68 (3.98-5.49)	2.78 (2.33-3.30)
**Marital status**			*P*<.001	*P*<.001	*P*<.001
	Never married	8098/12,322 (65.72)	1.00	1.00	1.00
	Married or living together as if married	1215/3309 (36.72)	0.28 (0.26-0.32)	0.32 (0.29-0.36)	0.56 (0.50-0.63)
	Divorced, widowed, or separated (and currently unmarried)	281/739 (38.02)	0.33 (0.27-0.39)	0.37 (0.31-0.44)	0.60 (0.50-0.74)
**Highest completed education level**			*P*<.001	*P*<.001	*P*<.001
	None or primary level	200/728 (27.47)	1.00	1.00	1.00
	Secondary level	8169/14,248 (57.33)	3.76 (3.07-4.62)	3.58 (2.89-4.43)	2.63 (2.14-3.22)
	Tertiary level	1225/1394 (87.88)	19.19 (14.17-25.99)	19.30 (14.15-26.34)	7.47 (5.45-10.22)
**Employment status**			*P*<.001	*P*<.001	*P*<.001
	Not in education or employment	3827/7917 (48.34)	1.00	1.00	1.00
	Informal employment	1544/2975 (51.9)	1.12 (1.00-1.26)	1.00 (0.89-1.13)	0.99 (0.87-1.12)
	In education	3656/4668 (78.32)	3.85 (3.46-4.28)	3.61 (3.24-4.01)	2.19 (1.97-2.44)
	Formal employment	567/810 (70)	2.47 (2.02-3.03)	2.07 (1.70-2.53)	1.55 (1.26-1.89)
**Time lived at current address (y)**			*P*<.001	*P*<.001	*P*<.001
	<2	2468/5473 (45.09)	1.00	1.00	1.00
	>2	7126/10,897 (65.39)	2.17 (1.98-2.37)	1.89 (1.72-2.07)	1.35 (1.21-1.51)
**Previous residence**			*P*<.001	*P*<.001	*P*<.001
	Always lived at this address	4406/6374 (69.12)	1.00	1.00	1.00
	Same suburb but different address	1867/3480 (53.65)	0.52 (0.46-0.59)	0.60 (0.54-0.68)	0.95 (0.82-1.09)
	Same town or city but different suburb	1902/3521 (54.02)	0.55 (0.49-0.63)	0.64 (0.56-0.72)	0.92 (0.79-1.07)
	Lived outside this town or city	1419/2995 (47.38)	0.43 (0.38-0.48)	0.49 (0.43-0.55)	0.70 (0.61-0.80)
**HIV status**			*P*=.002	*P*=.03	*P*=.78
	Negative	8952/15,168 (59.02)	1.00	1.00	1.00
	Positive	568/1076 (52.79)	0.75 (0.63-0.90)	0.82 (0.68-0.98)	0.97 (0.79-1.19)

^a^OR: odds ratio.

^b^Adjusted for age, sex, province, household wealth, marital status, highest completed education level, employment status, and time lived at current address and previous residence.

**Table 4:** Univariable and multivariable associations between exposure variables and social media use among youth recruited into a population-based survey in Zimbabwe (n=16,370).

Variable			Social media use, n (%)		Unadjusted OR^a^ (95% CI)		Age-, sex-, and province-adjusted OR (95% CI)		Final adjusted OR (95% CI)^b^
**Age (y)**					*P*=.86		*P*=.19		*P*<.001
	18-20	4843/8423 (57.5)		1.00		1.00		1.00	
	21-24	4617/7947 (58.1)		1.01 (0.94-1.08)		1.05 (0.98-1.13)		1.39 (1.27-1.52)	
**Sex**					*P*<.001		*P*<.001		*P*<.001
	Female	5078/9978 (50.89)		1.00		1.00		1.00	
	Male	4382/6392 (68.55)		2.03 (1.87-2.21)		2.05 (1.89-2.24)		1.41 (1.29-1.55)	
**Province**					*P*<.001		*P*<.001		*P*<.001
	Harare	3259/5557 (58.65)		1.00		1.00		1.00	
	Bulawayo	3349/5554 (60.3)		1.03 (0.92-1.16)		0.97 (0.87-1.09)		0.76 (0.67-0.85)	
	Mashonaland East	2852/5259 (54.23)		0.75 (0.65-0.86)		0.71 (0.62-0.82)		0.82 (0.72-0.92)	
**Household wealth index**					*P*<.001		*P*<.001		*P*<.001
	1 (poorest)	1366/3622 (37.71)		1.00		1.00		1.00	
	2	1631/3196 (51.03)		1.86 (1.65-2.11)		1.82 (1.61-2.05)		1.51 (1.34-1.72)	
	3	2053/3438 (59.71)		2.60 (2.30-2.94)		2.49 (2.21-2.81)		1.89 (1.67-2.14)	
	4	2402/3497 (68.69)		3.81 (3.33-4.35)		3.68 (3.24-4.17)		2.51 (2.21-2.85)	
	5 (richest)	2006/2610 (76.86)		6.04 (5.17-7.06)		5.71 (4.90-6.65)		3.45 (2.95-4.03)	
**Marital status**					*P*<.001		*P*<.001		*P*<.001
	Never married	7973/12,322 (64.71)		1.00		1.00		1.00	
	Married or living together as if married	1200/3309 (36.26)		0.29 (0.26-0.32)		0.29 (0.26-0.32)		0.50 (0.44-0.56)	
	Divorced, widowed, or separated (and currently unmarried)	287/739 (38.84)		0.37 (0.31-0.43)		0.37 (0.31-0.45)		0.60 (0.50-0.71)	
**Highest completed education level**					*P*<.001		*P*<.001		*P*<.001
	None or primary level	192/728 (26.37)		1.00		1.00		1.00	
	Secondary level	8042/14,248 (56.44)		3.75 (2.89-4.86)		3.56 (2.72-4.66)		2.59 (2.03-3.30)	
	Tertiary level	1226/1394 (87.95)		22.49 (16.27-31.08)		21.62 (15.48-30.19)		8.38 (6.06-11.57)	
**Employment status**					*P*<.001		*P*<.001		*P*<.001
	Not in education or employment	3792/7917 (47.9)		1.00		1.00		1.00	
	Informal employment	1536/2975 (51.63)		1.19 (1.07-1.31)		1.02 (0.92-1.14)		1.01 (0.91-1.13)	
	In education	3557/4668 (76.2)		3.49 (3.15-3.87)		3.34 (3.00-3.71)		1.97 (1.77-2.20)	
	Formal employment	575/810 (70.99)		2.71 (2.26-3.25)		2.21 (1.85-2.65)		1.62 (1.35-1.95)	
**Time lived at current address (y)**					*P*<.001		*P*<.001		*P*<.001
	<2	2499/5473 (45.66)		1.00		1.00		1.00	
	>2	6961/10,897 (63.88)		2.01 (1.85-2.18)		1.79 (1.65-1.95)		1.21 (1.09-1.35)	
**Previous residence**					*P*<.001		*P*<.001		*P*<.001
	Always lived at this address	4340/6374 (68.09)		1.00		1.00		1.00	
	Same suburb but different address	1848/3480 (53.1)		0.53 (0.47-0.60)		0.59 (0.53-0.66)		0.88 (0.77-1.01)	
	Same town or city but different suburb	1903/3521 (54.05)		0.58 (0.52-0.65)		0.65 (0.57-0.73)		0.89 (0.77-1.02)	
	Lived outside this town or city	1359/2995 (45.38)		0.41 (0.36-0.46)		0.45 (0.40-0.51)		0.59 (0.52-0.67)	
**HIV status**					*P*=.002		*P*=.02		*P*=.55
	Negative	8830/15,168 (58.21)		1.00		1.00		1.00	
	Positive	555/1076 (51.58)		0.75 (0.62-0.90)		0.80 (0.66-0.96)		0.94 (0.76-1.16)	

^a^OR: odds ratio.

^b^Adjusted for age, sex, province, household wealth, marital status, highest completed education level, employment status, and time lived at current address and previous residence.

### Relationships Between Mobile Phone Ownership, Internet Use, and Social Media Use

Individuals who owned a mobile phone had a 4.17 (95% CI 3.51-4.94; *P*<.001) times increase in the odds of using the internet (9342/15,454, 60.45%) compared to individuals who did not own a mobile phone (252/916, 27.51%). Similarly, individuals who owned a mobile phone had a 4.42 (95% CI 3.69-5.30; *P*<.001) times increase in the odds of using any social media (9227/15,454, 59.71%) compared to individuals who did not own a mobile phone (233/916, 25.44%).

## Discussion

### Principal Findings

Overall, this study demonstrates high levels of ownership of mobile phones among young people aged 18 to 24 years in urban and periurban areas of Zimbabwe. Nearly all these phones had both call and text functions, while 86.51% had WhatsApp and 63.55% had access to internet browsing on their device. This has important ramifications for the potential modalities for digital technology interventions, with the need for internet browsing likely to exclude a large proportion of potential users.

Where low levels of internet access are problematic, WhatsApp may potentially offer a compromise. Although WhatsApp may have reduced accessibility to young people compared to text messaging, this may be offset by less-stringent character-limit restrictions and reduced cost of message delivery if a project has Wi-Fi access, which may be more important considerations for an intervention. This may be particularly relevant where 2-way messaging with youth is required. However, if only “out” messaging to youth is needed, then text messaging may allow access to more individuals.

There were strong associations between household wealth and mobile phone ownership, internet use, and social media use, even after adjusting for a range of potential confounders. This highlights that the use of digital technologies may likely exacerbate inequality along socioeconomic lines. As a result, if these technologies are utilized, implementers must provide alternate strategies for individuals who do not have access. This could include loaning such digital technologies to individuals without access [17] or offering a nondigital alternative for engagement. In particular, choice of technology is crucial. For example, only a fifth of participants (3621/16,370, 22.12%) with a mobile phone accessed the internet every day or more often. Any technologies requiring regular internet access would therefore only be appropriate for this small subset of individuals.

In addition, although there was no association between sex and mobile phone ownership, male participants were more likely to use the internet or social media. Possible reasons include such technologies being considered “male” and being more socially acceptable for men to access. Future qualitative work is needed to explore this further. Importantly, the sex imbalance may indicate that such technologies are particularly appropriate to engage men, who are often considered “difficult” to reach and retain in services, particularly in relation to the HIV care cascade [18]. However, counter to this is that deployment of mHealth interventions may risk women being disenfranchised, unless provision can be made for this eventuality. Another interesting finding was the strong association between being married or previously married and not accessing the internet or social media. No association was found between marital status and mobile phone ownership. Social media use within marriage may affect quality of offline relationships, so married individuals may make a more active effort to disengage from social media [19]. Conversely, use of the internet and social media may also be seen as a means to seek out new relationships, perhaps contributing to higher levels of use in unmarried individuals.

In a survey of 634 young people aged 13 to 24 years in urban and periurban communities in Zimbabwe, Doyle et al [15] reported that 63% owned a phone, with a further 4.3% having access to a shared phone. Disaggregated by age, Doyle et al [15] found that 71.5% of the individuals aged 18 to 19 years and 84.7% of those aged 20 to 24 years owned a mobile phone. Our survey found higher proportions of mobile phone ownership of 83.22% (5657/6798) and 90.39% (9797/10,838) in these age groups, respectively. However, internet access was reported by 73.6% of individuals aged 18 to 19 years and 77.2% of those aged 20 to 24 years in the study by Doyle et al [15], in comparison to only 59.34% (3636/6127) and 58.17% (5958/10,243) in this study, respectively. This is despite this variable being restricted to individuals with access to a mobile phone in our study. Given that Doyle et al [15] collected data in 2018, it is expected that a higher proportion of individuals in these comparable age groups would own a mobile phone, due to increasing levels of access globally. However, it is less clear why there would be reduced levels of internet use. Possible reasons include variation between selected communities in both studies or differing case definitions. For example, the question related to internet use in our survey considered use generally, with options including never, once or twice a week, most days of the week, every day, or several times a day. However, Doyle et al [15] defined *internet access* as “if they reported accessing the internet once or more in the last 3 months, including on a device belonging to a family member or employer.” This broader definition may therefore have included some individuals who only access the internet occasionally in the Doyle et al [15] survey, but not our survey. Across both studies, mobile phone ownership was associated with increased age and higher education level, among young people in Zimbabwe, but not sex [15]. Additionally, both studies found that internet access was associated with male sex, increased age, higher education levels, and longer duration of residence at their current address [15].

Importantly, the strong associations between mobile phone ownership and internet or social media use suggest that mobile phones are a key entry point for use of other digital technologies. Similarly, among internet users, the vast majority of internet access was through a personal mobile phone. Together, this suggests that increasing smart phone ownership may allow for increased access to not only basic phone functions such as text and calls but also the internet. This may be a more cost-effective strategy to promote access to digital technologies compared to the provision of tablets or computers. It is also important to note that some digital interventions will simply not be deliverable by text or phone call, such as those involving videos, music, or peer-led social media communities [20-22]. However, access to technology alone is not sufficient. For example, 14.83% (1456/9821) of those who owned mobile phones capable of accessing the internet did not regularly use the internet. Identification of the additional barriers faced by these individuals in accessing the internet is key, determining if this was an active choice or if other factors such as cost of data were prohibitive. There is also a need to go beyond simply investigating where disparities exist. There needs to be an active effort to address any disparities in order to build digital readiness at a community level [23]. This will require significant investment in local, national, and international infrastructure, alongside lowering the individual barriers to access such as the cost of hardware and internet access [15,23]. Economic evaluations on the potential benefit of subsidies for smartphones or communal Wi-Fi networks would be prudent to consider whether such interventions may be cost-effective. One must also consider the possibility that digital technologies may not be the way forward in all settings. In addition to the expense required to upgrade current infrastructure, it may not be acceptable or feasible to local populations. Within the CHIEDZA trial, a substudy assessed the use of a novel application called ITHAKA to support HIV self-testing among youth, either on a tablet on site or on their mobile phone off-site. However, only 5.8% (128/2181) youth opted for HIV self-testing over provider testing. Furthermore, of those who performed HIV self-testing off-site, less than half (9/19, 47%) completed their testing journey [24]. Although some issues were technology focused, such as lack of phone ownership, limited functionality, and erratic network coverage, other factors included a lack of agency or private space and a desire for provider support during testing. As a result, even addressing the digital factors may not have led to acceptability and feasibility.

### Strengths and Limitations

Strengths of this study include a population-based sample with a very large sample size, random sampling, and the fact that digital technologies were not required to access the study. Limitations include the potential for social desirability bias, especially given that mobile phones may be perceived as a symbol of wealth and status. Of note, 151 mobile phone owners had phones that could not call or text. Of them, 110 (72.8%) were also neither equipped to use WhatsApp or browse the internet. It is interesting that these individuals still considered themselves mobile phone owners, despite their phones not having any of the expected core functionalities. Social desirability bias may have influenced this self-classification. Although we were able to construct a household wealth index from 6 household assets, the reluctance of some participants to disclose information related to estimated household income demonstrates the difficulties related to collecting data on wealth. Importantly, we are unable to demonstrate causality from this cross-sectional study. Therefore, although we hypothesized that, for example, being wealthier increases the odds of owning a phone or accessing the internet, the inverse may account for at least some of the relationship. For example, owning a mobile phone may allow a young adult to engage more dynamically with business opportunities, thus increasing wealth. Selection bias may also have influenced the results. Of the 1039 individuals enumerated but not enrolled, 696 (67%) declined participation and a further 77 (7.4%) were not found. These participants may have different characteristics from those enrolled. Finally, another key limitation is generalizability, with all participants recruited from urban or periurban areas, which are likely to have different levels of access to digital technologies compared to rural areas.

### Conclusions

To our knowledge, this is the largest population-based survey examining the factors associated with digital technology use among young people in Africa. It demonstrates that a high proportion of young adults in the urban or periurban areas studied own or at least have access to a mobile phone. However, to fully make use of digital technology in Zimbabwe, more effort must be made to increase access further, targeted at those with lower levels of wealth, in order that interventions do not exacerbate existing socioeconomic inequality. Before the implementation of digital technology–based interventions, whether urban or rural, a key component of formative work must be focused on the use of and accessibility to the required technologies.

## Appendix

Multimedia Appendix 1 Survey questions and response categories.

Multimedia Appendix 2 Construction of wealth index using principal component analysis.

Multimedia Appendix 3 WhatsApp and internet use according to phone functionality among mobile phone owners.

Multimedia Appendix 4 Univariable associations between age, education level, and time at current address and mobile phone ownership, internet use, and social media use.

## Abbreviations

aOR: adjusted odds ratio
CONSORT: Consolidated Standards for Reporting Trials
mHealth: mobile health

## Data Availability

The datasets used and analyzed during this study are available from the corresponding author on reasonable request. Additionally, individual, anonymized participant data and a data dictionary will be available through The London School of Hygiene & Tropical Medicine repository (Data Compass) 12 months after the publication of results.
