# Supplementary material for: Factors Associated With the Use of Digital Technology Among Youth in Zimbabwe: Findings From a Cross-Sectional Population-Based Survey
Source: J Med Internet Res. 2024 Sep 23;26:e52670. doi: 10.2196/52670 (PMC11459104; doi:10.2196/52670)
Supplement: Multimedia Appendix 1 [file jmir_v26i1e52670_app1.docx]

| Question | Response categories |
| --- | --- |
| Sociodemographic | |
| How old are you?  *Calculate age in completed years – must be between 18-24years* |  |
| How do you identify yourself? (select one) | - Male - Female - Non-binary - Transgender |
| What is the sex you were assigned to at birth i.e. what is your biological sex? (select one) | - Male - Female - Intersex |
| How long have you lived in this current address? (select one) | - Less than 12 months - 12 months to 24 months (2 years) - >2 years to 3 years - More than 3 years |
| Thinking about where you lived before you moved to this address, which one applies (select one): | - I have always only lived at this address - I lived in the same suburb, but different address - I lived in the same town or city but not in the same suburb - I lived outside this town/city |
| What would you say your average regular household income (in United States Dollar equivalent) would be, per month? (select one)  *A household defined as person or group of related or unrelated persons who live together in the same dwelling or unit(s) of a dwelling, who acknowledged one adult male or female as head of the household, who share the same housekeeping arrangements, and who are considered a single unit* | - Less than USD 50 - USD50-100 - USD101-200 - USD 201-500 - USD 501-900 - More than USD 900 - Don’t know/Don’t want to say |
| Does your household have the following working items? (Yes/No for each option) | - Fridge (Yes/No) - Bicycle (Yes/No) - Car/truck (Yes/No) - Television (Yes/No) - Radio (Yes/No) - Microwave (Yes/No) - Cell phone (Yes/No) - Computer or laptop or tablet (Yes/No) |
| What is the highest level of education you have completed? (select one)  *If still in education, tick the highest level completed: e.g. if currently in Secondary Form 6, then the highest completed is Secondary Form 5* | - Never attended school - Primary Grade 1 - Primary Grade 2 - Primary Grade 3 - Primary Grade 4 - Primary Grade 5 - Primary Grade 6 - Primary Grade 7 - Secondary Form 1 - Secondary Form 2 - Secondary Form 3 - Secondary Form 4 - Secondary Form 5 - Secondary Form 6 - Vocational / trade school - College (diploma, certificate) - University |
| Currently, what is the main activity you are engaged in? (select one)  *Employed defined as having own registered business or work in the past seven days that generates a regular income. Includes not having worked in the past seven days but are regularly employed but were absent from work for leave, illness, vacation, or other such reason. Informal sector defined as not having a “regular” income.* | - In education (university/college/school) - Owns a registered business or has a formal job that pays a wage i.e. employed - Work in informal sector (e.g. subsistence farming, informal income-generating activities) - None of the above |
| What is your marital status? (select one) | - Married or living together as if married - Never married - Divorced, widowed or separated (and currently unmarried) |
| Use digital technology | |
| Which of the following best describes your situation regarding mobile/cell phones? (select one) | - I have my own cell phone - I share the cell phone of a family member - I share someone else’s cell phone (not a family member) - I do not use a cell phone |
| If you own your own cell phone, which of the following is possible to do on your phone? (Yes/No for each option) | - Calls (Yes/No) - Text or SMS messaging (Yes/No) - Whatsapp (Yes/No) - Internet browsing (Yes/No) - Not applicable, I do not have my own cell phone (Yes/No) |
| How often do you use the internet? (select one)  *Internet use defined as using the internet on any type of device (e.g. phone, table, computer) for any reason including social media except Whatsapp* | - Never - Once or twice a week - Most days of the week - Everyday - Several times a day |
| Where do you most often use the internet? (select one) | - My phone - Someone else’s phone - On my personal computer or other device (e.g. tablet) - On a computer or other device at home (that doesn’t only belong to me and is also used by others) - At an internet café - At work - At school - Other [Specify] _______________________________________ - I don’t use the internet |
| Which social media sites do you use regularly? (Yes/No for each option) | - WhatsApp (Yes/No) - Facebook (Yes/No) - Instagram (Yes/No) - Twitter (Yes/No) - Snapchat (Yes/No) - YouTube (Yes/No) - TikTok (Yes/No) - LinkedIn (Yes/No) - Other [Specify] _______________________________________ (Yes/No) - Don’t use any social media (Yes/No) |
