# Supplementary material for: Factors Associated With the Use of Digital Technology Among Youth in Zimbabwe: Findings From a Cross-Sectional Population-Based Survey
Source: J Med Internet Res. 2024 Sep 23;26:e52670. doi: 10.2196/52670 (PMC11459104; doi:10.2196/52670)
Supplement: Multimedia Appendix 2 [file jmir_v26i1e52670_app2.docx]

We created household wealth indicators and subsequently categorised these into wealth quintiles using Principal Component Analysis (PCA), which involved four steps in Stata. PCA is a statistical technique that helps to identify patterns in data based on the correlation between variables, thereby reducing the dimensionality of the observations while retaining most of the variation in the observations.

**Step 1: Prepare the household assets variables to binary and coded 0/1**

Six household assets were used: refrigerator, bicycle, car or truck, television, radio, and microwave. These were binary variables (0/1) for each asset, where 1 indicates ownership of the asset and 0 indicates absence

**Step 2: Conduct Principal Component Analysis (PCA)**

We use the **pca** command in Stata. This command performs PCA and retains the principal components (PCs) of the data.

*pca fridge bicycle car television radio microwave, components(1)*

The option **components(1)** tells Stata to retain only the first principal component, which typically captures the largest portion of the variance among the asset indicators.

1. We examined the output: The Stata output provided eigenvalues, the proportion of variance explained by each principal component, and the component loadings (coefficients) for each asset variable on each principal component (Table S1).

| Table S1a: Proportion of variance explained by each principal component | | | | |
| --- | --- | --- | --- | --- |
| **Component** | **Eigenvalue** | **Difference** | **Proportion** | **Cumulative** |
| *Comp1* | 2.325 | 1.300 | 0.388 | 0.388 |
| *Comp2* | 1.024 | 0.205 | 0.171 | 0.558 |
| *Comp3* | 0.820 | 0.099 | 0.137 | 0.695 |
| *Comp4* | 0.721 | 0.085 | 0.120 | 0.815 |
| *Comp5* | 0.636 | 0.162 | 0.106 | 0.921 |
| *Comp6* | 0.473 |  | 0.079 | 1.000 |

| Table S1b: Principal components (eigenvectors) | | |
| --- | --- | --- |
| ***Variable*** | **Comp1** | **Unexplained** |
| fridge | 0.479 | 0.467 |
| bicycle | 0.241 | 0.865 |
| car | 0.380 | 0.663 |
| tv | 0.461 | 0.506 |
| radio | 0.409 | 0.612 |
| microwave | 0.434 | 0.562 |

1. The first principal component was used to create a wealth index because it explained the largest portion of the variance in the asset variables. The decision on the number of components to retain was also informed by the eigenvalue-one criterion (Kaiser criterion) or a scree plot (Table S2 and Figure S1).

*screeplot, yline(1) ci(het)*

**Figure S1**: Scree plot of eigenvalues

*estat kmo*

| Table S2: Kaiser-Meyer-Olkin measure of sampling adequacy | |
| --- | --- |
| **Variable** | **KMO** |
| Fridge | 0.7154 |
| Bicylce | 0.7750 |
| Car | 0.7605 |
| Tv | 0.6873 |
| Radio | 0.7750 |
| Microwave | 0.7700 |
| **Overall** | **0.7363** |

**Step 3: Generate Wealth Index Scores**

After performing PCA, we used the **predict** command to generate wealth index scores based on the first principal component. These scores are the households' coordinates along the first principal component.

*predict wealth_index, score*

This command creates a new variable, **wealth_index**, which contains the wealth scores for each household.

| Table S3: Scoring coefficients | |
| --- | --- |
| **Variable** | **Component1 loadings** |
| Fridge | 0.4788 |
| Bicycle | 0.2412 |
| Car | 0.3805 |
| Tv | 0.4609 |
| Radio | 0.4087 |
| Microwave | 0.4340 |

Component loadings shown in Table S3 displays how each asset contributes to the wealth index. Higher absolute values indicate a stronger contribution to the wealth score

**Step 4: Create Wealth Quintiles**

To divide the **wealth_index** into quintiles, we used the **xtile** command. This categorised households into 5 groups based on their wealth index scores, from the poorest (1st quintile) to the wealthiest (5th quintile).

*xtile wealth_quintiles = wealth_index, nq(5)*

This command generates a new variable, **wealth_quintiles**, which indicates the wealth quintile of each household.

**Note**: Words in italics are Stata command.
