# Supplementary material for: Factors Associated With the Use of Digital Technology Among Youth in Zimbabwe: Findings From a Cross-Sectional Population-Based Survey
Source: J Med Internet Res. 2024 Sep 23;26:e52670. doi: 10.2196/52670 (PMC11459104; doi:10.2196/52670)
Supplement: Multimedia Appendix 3 [file jmir_v26i1e52670_app3.docx]

|  | WhatsApp possible on phone | |  |
| --- | --- | --- | --- |
| WhatsApp used regularly | *No* | *Yes* | *Total* |
| *No* | 1732 (83.07%) | 184 (1.38%) | 1916 (12.40%) |
| *Yes* | 353 (16.93%) | 13185 (98.62%) | 13538 (87.60%) |
| *Total* | 2085 (100.00%) | 13369 (100.00%) | 15454 (100.00%) |

Table S1: Regular WhatsApp use according to whether WhatsApp possible on phone, among mobile phone owners

Table S2: Internet use according to whether internet access possible on phone, among mobile phone owners

|  | Internet access possible on phone | |  |
| --- | --- | --- | --- |
| Internet accessed | *No* | *Yes* | *Total* |
| *No* | 4656 (82.66%) | 1456 (14.83%) | 6112 (39.55%) |
| *Yes* | 977 (17.34%) | 8365 (85.17%) | 9342 (60.45%) |
| *Total* | 5633 (100.00%) | 9821 (100.00%) | 15454 (100.00%) |
