# Supplementary material for: Factors Associated With the Use of Digital Technology Among Youth in Zimbabwe: Findings From a Cross-Sectional Population-Based Survey
Source: J Med Internet Res. 2024 Sep 23;26:e52670. doi: 10.2196/52670 (PMC11459104; doi:10.2196/52670)
Supplement: Multimedia Appendix 4 [file jmir_v26i1e52670_app4.docx]

| Variable | Mobile phone ownership  N (%)  [N = 17636] | Internet usage  N (%)  [N = 16370] | Social media usage  N (%)  [N = 16370] |
| --- | --- | --- | --- |
| Age (y) |  |  |  |
| 18 | 3427/4234 (80.94%) | 2182/3746 (58.25%) | 2062/3746 (55.05%) |
| 19 | 2230/2564 (86.97%) | 1454/2381 (61.07%) | 1398/2381 (58.71%) |
| 20 | 2184/2441 (89.47%) | 1395/2296 (60.76%) | 1383/2296 (60.24%) |
| 21 | 2036/2249 (90.53%) | 1278/2123 (60.20%) | 1285/2123 (60.53%) |
| 22 | 1689/1878 (89.94%) | 1026/1764 (58.16%) | 1036/1764 (58.73%) |
| 23 | 1703/1886 (90.30%) | 982/1791 (54.83%) | 1008/1791 (56.28%) |
| 24 | 2185/2384 (91.65%) | 1277/2269 (56.28%) | 1288/2269 (56.77%) |
| Highest completed education level |  |  |  |
| None or primary level | 647/867 (74.63%) | 200/728 (27.47%) | 192/728 (26.37%) |
| Secondary level 1 - 4 | 10850/12638 (85.85%) | 5972/11594 (51.51%) | 5851/11594 (50.47%) |
| Secondary level 5 – 6 | 2582/2725 (94.75%) | 2197/2654 (82.78%) | 2191/2654 (82.55%) |
| Vocational/trade school | 211/220 (95.91%) | 177/217 (81.57%) | 170/217 (78.34%) |
| College (diploma, certificate) | 556/569 (97.72%) | 487/565 (86.19%) | 480/565 (84.96%) |
| University | 608/617 (98.54%) | 561/612 (91.67%) | 576/612 (94.12%) |
| Time lived at current address |  |  |  |
| Less than 12 months | 3614/4241 (85.22%) | 1636/3892 (42.03%) | 1693/3892 (43.50%) |
| 12 to 24 months | 1485/1701 (87.30%) | 832/1581 (52.62%) | 806/1581 (50.98%) |
| 24 to 36 months | 1442/1685 (85.58%) | 844/1549 (54.49%) | 799/1549 (51.58%) |
| More than 36 months | 8913/10009 (89.05%) | 6282/9348 (67.20%) | 6162/9348 (65.92%) |
